# *EGFR* C797S, *EGFR* T790M and *EGFR* sensitizing mutations in non-small cell lung cancer revealed by six-color crystal digital PCR

**DOI:** 10.18632/oncotarget.26446

**Published:** 2018-12-21

**Authors:** Jordan Madic, Cécile Jovelet, Julien Lopez, Barbara André, Jean Fatien, Isabelle Miran, Aurélie Honoré, Laura Lezquita, Benjamin Besse, Ludovic Lacroix, Magali Droniou

**Affiliations:** ^1^ Stilla Technologies, 1 Mail du Professeur Georges Mathé, Villejuif, France; ^2^ Plateforme de Génomique, Module de Biopathologie Moléculaire et Centre de Ressources Biologiques, AMMICa, INSERM US23/CNRS UMS3655, Gustave Roussy, Villejuif, France; ^3^ Ecole Polytechnique, Route de Saclay, Palaiseau, France; ^4^ Département de Biologie et Pathologie Médicales, Institut Gustave Roussy, Villejuif, France; ^5^ Département d’Oncologie Médicale, Gustave Roussy, Villejuif, France

**Keywords:** *EGFR*, multiplexing, monitoring, NSCLC, digital PCR

## Abstract

**Background:**

Detection of *EGFR* sensitizing and p.T790M and p.C797S resistance mutations is particularly important for non-small cell lung cancer (NSCLC) patient therapy management. Non-invasive blood-based monitoring of these mutations may pave the way to a fine-tuned personalized treatment. Digital PCR has emerged as an extremely sensitive method to detect rare mutations, however its major limitation is the number of hotspots that can be simultaneously differentiated.

**Methods:**

We developed a 6-color digital PCR assay for the detection and quantification of 19 most prevalent *EGFR* sensitizing and resistance mutations and evaluated this assay on 82 tumor and plasma samples from NSLC patients.

**Results:**

Limits of detection (LOD) for the 6-color digital PCR assay were assessed on serial dilutions of DNA standards. We found that the 6-color assay enabled detection of mutant fractions as low as 1 mutant in 1025 wild-type molecules, depending on the mutation targeted, when assayed in a background of 10 000 wild-type DNA copies. *EGFR* mutant allelic fraction was also measured on tumor and plasma samples by 6-color digital PCR, and displayed a highly significant correlation with next generation sequencing and 3-color digital PCR. Lastly, the 6-color digital PCR assay was performed on several longitudinal plasma samples from four patients and revealed levels of sensitizing and resistance *EGFR* mutations that reflected well the course of the disease.

**Conclusion:**

This 6-color Crystal digital PCR assay could represent a robust solution using digital PCR for the monitoring of *EGFR* mutations.

## INTRODUCTION

Non-small cell lung cancer (NSCLC) accounts for 85% to 90% of lung cancers and is the leading cause of cancer-related mortality worldwide [1, 2]. Dysregulation of the epidermal growth factor receptor (*EGFR*) by activating mutations is a well-known oncogenic mechanism in this pathology. In-Frame deletion-insertions (or delins) in exon 19, substitutions of a leucine for an arginine at codon 858 (p.L858R) or for a glutamine at codon 861 (p.L861Q) and glycine change for an alanine, a cysteine or a serine at codon 719 (p.G719A, p.G719C, p.G719S) account for more than 90% of *EGFR* activating mutations [3, 4]. Targeted therapy aiming at these mutations using tyrosine kinase inhibitors (TKIs) has greatly improved the treatment of NSCLC compared to classical platinum-based chemotherapy mutations [5]. However, most of TKIs-responder patients eventually develop resistance with more than 50% of this subset of patients carrying the exon 20 p.T790M mutation [6]. Third generation TKIs have been developed to circumvent the T790M-mediated resistance to targeted therapy but despite good initial clinical response in prospective cohorts, several resistance mechanisms have been reported, among which the p.C797S point mutation in exon 20 [7, 8, 9, 10, 11]. The p.C797S mutation was observed in 22% to 40% of the patients treated with osimertinib, a FDA-approved third generation TKI, and was also reported to mediate resistance to other third generation TKIs [10, 12, 13, 14, 15]. These observations highlight the importance of profiling *EGFR* mutations to predict clinical response to *EGFR* TKIs and provide an effective therapeutic regimen. In clinical settings, tumor DNA obtained from malignant tissue is the major source for *EGFR* genotyping while circulating cell-free DNA (cfDNA) can be used when biopsy is not feasible [16]. Several molecular methods based on real-time PCR, digital PCR and next generation sequencing have been developed to achieve *EGFR* genetic profiling [17]. Digital PCR is a promising approach by many aspects as it combines a high sensitivity, an absolute quantification and a short turnaround time [17]. Currently, *EGFR* mutation profiling in clinical settings requires scanning a few mutation hotspots to predict *EGFR*-TKIs treatment efficacy. To enable the simultaneous detection of several *EGFR* mutations in one reaction, despite the limited number of fluorescent channels available in existing digital PCR systems, alternative multiplex digital PCR strategies have been developed [18, 19]. However, these methods, based on varying fluorescent probe concentrations, generate fluorescent partition populations that may be difficult to differentiate in presence of high quantity of input DNA or poor DNA quality [19]. In this study, we have developed a six-color digital PCR assay for the detection of the most prevalent *EGFR* mutations in NSCLC in a single reaction, based on the Naica system, for which an alternative reading and analysis workflow was developed. Using 6 fluorescence detection channels, in-frame deletion-insertions in *EGFR* exon 19 could be differentiated from 5 other sensitizing mutations, as well as the p.T790M and the p.C797S resistance mutations. This assay was optimized and evaluated in tumor and cfDNA samples from NSCLC patients.

## RESULTS

### Determination of the limit of blank and the limit of detection for the 6-color digital PCR *EGFR* assay

A limit of blank (LOB) was calculated for each of the detection channels allocated to a target as follows: FAM and Atto700 for *EGFR* exon 19 in-frame delins, Cyanine 5 for *EGFR* p.L858R, p.L861Q, p.G719A, p.G719C and p.G719S activating mutations, Cyanine 3 and ROX for *EGFR* p.T790M and *EGFR* p.C797S resistance mutations respectively. A total of N = 34 six-color digital PCR experiments containing on average 11156 ± 1731.8 copies of wild-type DNA per 25 μl reaction were performed. The LOB with a confidence level (1− α) was defined as the maximum number of false positive partitions that are plausible with a 1− α probability (here 95% for risk α = 5%). The number of false positive events was recorded in each of the targeted channel of detection and the mean μ and the standard deviation σ of the false positive distribution were calculated, then corrected per detection channel using the following formula: μ_corr_ = μ +1.645 σ√N where N is the number of experiments performed. The LOB with 95% confidence level in number of false-positives partitions per reaction was determined by fitting the calculated μ_corr_ on Normal Law approximation and Chernoff's inequality. The LOB was set as 3 false-positive partitions for exon 19 delins, 4 false-positive partitions for the T790M and the C797S mutation and 5 false-positive partitions for all the other mutations. The theoretical limits of detection with 95% confidence level were calculated for each group of mutations according to Milbury et al. [20].

### Six-color digital PCR *EGFR* assay sensitivity

**Figure 1:**
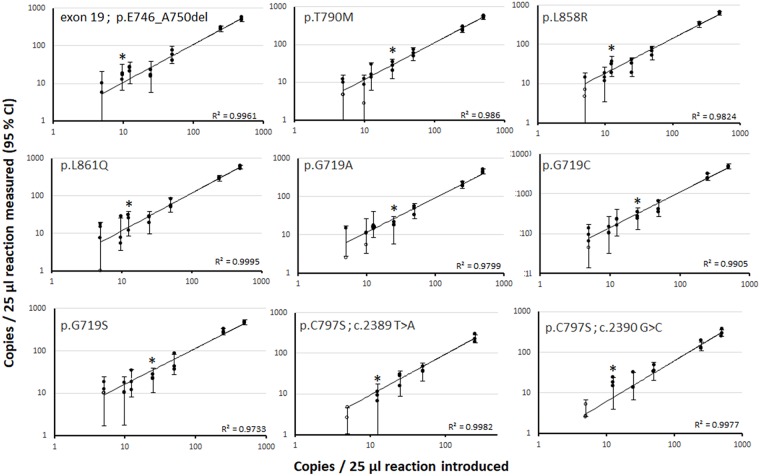
Evaluation of the six-color digital PCR assay sensitivity on serial dilution of mutant DNAs in a background of wild-type DNA Nine separate serial dilutions of mutant DNA standards harboring the 9 *EGFR* targeted mutations were constituted and tested in six-color digital PCR experiments. The 95% confidence interval (CI) was derived from the mean of the theoretical 95% CI obtained for each replicate and based on the Poisson uncertainty. Black circles represent the result of each replicate experiment. Empty circles represent the values that are below the limit of blank (LOB). Asterisks indicate the empirically-determined LOD for each mutation tested.

Six-color digital PCR *EGFR* assay sensitivity was assayed on 9 serial dilutions of mutant genomic DNA, each bearing one of the following mutations: exon 19 in-frame delins, p.G719A, p.G719C, p.G719S, p.L858R, p.L861Q, p.T790M, p.C797S c.2389 T>A and p.C797S c.2390 G>C (Figure 1). All DNA reference material were purchased from commercial providers and tested using single plex digital PCR prior to 6-color assay sensitivity monitoring. For all the mutations except p.C797S c.2389 T>A and p.C797S c.2390 G>C, mutant DNA targets were serially diluted from 500 to 5 copies per μl in a constant background of 10^4^ copies of wild-type DNA per μl, to achieve mutant allele fractions (MAF) of 5%, 2.5%, 0.5%, 0.25%, 0.125%, 0.0975% and 0.05%. For p.C797S c.2389 T>A, the expected MAFs were 2.5%, 0.5%, 0.25% 0.125% and 0.05%, and for p.C797S c.2390 G>C, 5%, 2.5%, 0.5%, 0.25% 0.125% and 0.05%. One microliter of each dilution was assayed in triplicate, except for dilutions containing 5 mutant copies per μl, which were performed in quadruplicate. The coefficients of determination of linear regressions performed between introduced and measured of copies per reaction ranged from R^2^ = 0.9733 to R^2^ = 0.9995 depending on the mutation targeted. We considered as positive any MAF values with at least two replicates in which the number of positive partitions was equivalent or higher than the theoretical limit of detection (LOD). Limit of detection for the p.C797S c.2389 T>A and p.C797S c.2390 G>C was thus empirically characterized as an average of 12.5 copies per 25μl reaction, corresponding to a MAF of 0.125%. The exon19 delins detection system enabled the detection of a MAF of 0.0975%. The limit of detection for *EGFR* resistance mutation p.T790M yielded a MAF of 0.25%, while activating mutations in the Cy5 detection channel were detected with MAFs of 0.125-0.25%.

### Detection of *EGFR* mutations in NSCLC patients

A cohort of 82 patient samples for which the *EGFR* mutational status was determined by NGS were included in the study. A total of 33 extracts of tumor DNA derived from 21 frozen and 12 FFPE tissue samples were analyzed by six-color digital PCR and NGS (Table 1). Among the 24 *EGFR* mutated tumor samples, both methods detected *EGFR* exon 19 delins in 14 samples (58%). Six-color digital PCR identified 10 samples positive for *EGFR* sensitizing mutations, which were characterized as *EGFR* p.L858R and p.L861Q mutations in 9 samples (31%) and EGFR p.G719S in 1 sample (3%) by NGS. *EGFR* p.T790M mutation was found in 13 samples (45%) by digital PCR and 12 samples (41%) by NGS. Finally, *EGFR* p.C797S mutation was found in 5 samples (17%) by both methods. Nine FFPE samples were found *EGFR* wild-type by both NGS and 6-color digital PCR. Regarding the analysis of cfDNA, 49 DNA samples were assayed by six-color digital PCR and NGS (Table 1). A total of 1.8 ng from 139 ng of cfDNA were assayed using 6-color dPCR while NGS was performed on 10 ng of cfDNA. Cell-free circulating DNA concentrations were higher than that of healthy individual [20], ranging from 2.8 to 308 ng per mL of plasma (median: 28 ng per mL), representing 862 to 93333 cfDNA copies per mL of plasma (median: 8485 copies per mL). *EGFR* exon 19 delins were detected by both NGS and six-color digital PCR in 24 samples (69%). Six-color digital PCR detected the presence of *EGFR* sensitizing mutations in 11 samples, which were identified as EGFR p.L858R for 9 samples (26%), and *EGFR* p.G719A and p.G719C in 2 samples (4%) by NGS. In plasma samples bearing an *EGFR* activating mutation, 6-color digital PCR also detected 14 *EGFR* T790M mutations (37%) whereas NGS detected 13 *EGFR* T790M mutations (34%). Both 6-color digital PCR and NGS detected *EGFR* p.C797S mutation in 3 T790M positive samples (9%). The 14 remaining cfDNA samples were found *EGFR* wild-type by both NGS and 6-color digital PCR.

**Table 1:** Determination of mutant allele fraction by 6-color digital PCR, NGS and 3-color digital PCR in tumor and plasma of metastatic NSCLC patients

						Mutant allele fraction by 3-color dPCR (%)		Mutant allele fraction by 6-color dPCR (%)				Mutant allele fraction by NGS (%)			
Case no.	Sampling time (day)	Sample type	EGFR mutational status by NGS	Exon 19 genomic variant	cfDNA (ng/ml plasma)	Activating mutations	T790M	E19 delins	L858R L861Q G719X	T790M	C797S	E19 delins	L858R L861Q G719X	T790M	C797S
1	NA	Frozen tumor	E19 delins	c.2251_2262del	NA	-	-	37.4	0	0	0	32.7	0	0	0
2	NA	Frozen tumor	E19 delins	c.2236_2250del	NA	-	-	21.6	0	2.8	0	25.6	0	0	0
3	NA	Frozen tumor	E19 delins, T790M	c.2235_2249del	NA	-	-	60.7	0	33.5	0	58.2	0	57.6	0
4	NA	Frozen tumor	E19 delins	c.2235_2249del	NA	-	-	56.6	0	0	0	55.1	0	0	0
5	NA	Frozen tumor	E19 delins, T790M	c.2236_2248delinsC	NA	-	-	57.7	0	9.7	0	74.1	0	15.0	0
6	NA	Frozen tumor	L861Q	NA	NA	-	-	0	24.3	0	0	0	31.1	0	0
7	NA	Frozen tumor	L858R	NA	NA	-	-	0	11.7	0	0	0	12.6	0	0
8	NA	Frozen tumor	L861Q	NA	NA	-	-	0	9.3	0	0	0	9.4	0	0
9	NA	Frozen tumor	G719S, T790M	NA	NA	-	-	0	26.7	8.2	0	0	36.4	11.6	0
10	NA	Frozen tumor	L858R	NA	NA	-	-	0	48.6	0	0	0	79.3	0	0
11	NA	Frozen tumor	E19 delins	c.2234_2245del	NA	-	-	36.2	0	0	0	35.3	0	0	0
12	NA	Frozen tumor	E19 delins	c.2239_2247del	NA	-	-	38.3	0	0	0	45.8	0	0	0
13	NA	Frozen tumor	L858R, T790M	NA	NA	-	-	0	28.4	12.5	0	0	36.5	18.1	0
14	NA	Frozen tumor	E19 delins, T790M	c.2236_2250del	NA	-	-	17.7	0	4.8	0	19.8	0	4.8	0
15	NA	Frozen tumor	E19 delins, T790M	c.2236_2250del	NA	-	-	69.3	0	7.0	0	77.8	0	10	0
16	NA	Frozen tumor	L858R, T790M	NA	NA	-	-	0	41.6	9.3	0	0	62.1	11.4	0
17	NA	Frozen tumor	L858R, T790M, C797S	NA	NA	-	-	0	38.6	10.7	11.0	0	63	14	16.2
18	NA	Frozen tumor	E19 delins, T790M, C797S	c.2239_2248delinsC	NA	-	-	36.4	0	13.4	17.6	36.3	0	6.7	18.1
19	NA	Frozen tumor	E19 delins, T790M, C797S	c.2235_2249del	NA	-	-	41.2	0	5.2	6.4	38.9	0	10.1	15
20	NA	Frozen tumor	E19 delins, T790M, C797S	c.2235_2249del	NA	-	-	98.6	0	15.2	87.8	98.7	0	14.5	11.8
21	NA	Frozen tumor	E19 delins, T790M, C797S	c.2235_2249del	NA	-	-	79.0	0	37.5	44.1	77.7	0	63.9	17.6
22	NA	FFPE	L858R	NA	NA	-	-	0	41.3	0	0	0	69.6	0	0
23	NA	FFPE	L858R	NA	NA	-	-	0	32.5	0	0	0	53.7	0	0
24	NA	FFPE	E19 delins	c.2239_2256del	NA	-	-	90.2	0	0	0	38	0	0	0
25	NA	FFPE	EGFR WT	NA	NA	-	-	0	0	0	0	0	0	0	0
26	NA	FFPE	EGFR WT	NA	NA	-	-	0	0	0	0	0	0	0	0
27	NA	FFPE	EGFR WT	NA	NA	-	-	0	0	0	0	0	0	0	0
28	NA	FFPE	EGFR WT	NA	NA	-	-	0	0	0	0	0	0	0	0
29	NA	FFPE	EGFR WT	NA	NA	-	-	0	0	0	0	0	0	0	0
30	NA	FFPE	EGFR WT	NA	NA	-	-	0	0	0	0	0	0	0	0
31	NA	FFPE	EGFR WT	NA	NA	-	-	0	0	0	0	0	0	0	0
32	NA	FFPE	EGFR WT	NA	NA	-	-	0	0	0	0	0	0	0	0
33	NA	FFPE	EGFR WT	NA	NA	-	-	0	0	0	0	0	0	0	0
34	NA	Plasma	E19 delins	c.2235_2249del	24	-	-	1.1	0	0	0	0.5	0	0	0
35	NA	Plasma	G719C	NA	78.7	-	-	0	7.3	0	0	0	5.8	0	0
36	NA	Plasma	E19 delins, T790M	c.2239_2251delinsC	18.7	-	-	5.9	0	6.6	0	1.7	0	2.6	0
37	NA	Plasma	E19 delins,	c.2235_2246del	184	2.4	0.02	3.2	0	0.05	0	2.5	0	0	0
38	NA	Plasma	E19 delins, T790M	c.2235_2249del	32	6.9	2.1	6.5	0	3.3	0	10.4	0	3.5	0
39	NA	Plasma	E19 delins, T790M	c.2235_2249del	34.7	-	-	53.9	0	7.7	0	65.1	0	14.4	0
40	NA	Plasma	L858R	NA	8	-	-	0	7.1	0	0	0	3	0	0
41	NA	Plasma	L858R, T790M	NA	14.7	6.7	0.8	0	5.9	1.9	0	0	3.7	1.8	0
42	NA	Plasma	L858R	NA	88.3	-	-	0	2.6	0	0	0	0.7	0	0
43	NA	Plasma	L858R. T790M	NA	16	14.4	2.9	0	11.3	1.7	0	0	12.1	2.1	0
44	NA	Plasma	E19 delins	c.2236_2250del	18.3	-	-	2.8	0	0	0	4.4	0	0	0
45	NA	Plasma	E19 delins	c.2235_2249del	30	-	-	6.5	0	0	0	5.3	0	0	0
46	NA	Plasma	L858R	NA	62	-	-	0	14.8	0	0	0	9.9	0	0
47	NA	Plasma	G719A	NA	29.3	-	-	0	22.2	0	0	0	25.2	0	0
48	NA	Plasma	L858R	NA	19.6	9.8	0	0	10.1	0	0	0	11.9	0	0
49	NA	Plasma	E19 delins	c.2239_2256del	12.9	29.1	0	38.7	0	0	0	44.7	0	0	0
50	NA	Plasma	E19 delins	c.2237_2253delinsTTGCT	12.1	34.4	0	31.4	0	0	0	37.2	0	0	0
51	NA	Plasma	EGFR WT	NA	4	0	0	0	0	0	0	0	0	0	0
52	NA	Plasma	EGFR WT	NA	3.7	0	0	0	0	0	0	0	0	0	0
53	NA	Plasma	EGFR WT	NA	25.3	0	0	0	0	0	0	0	0	0	0
54	NA	Plasma	EGFR WT	NA	16.2	0	0	0	0	0	0	0	0	0	0
55	NA	Plasma	EGFR WT	NA	4.1	0	0	0	0	0	0	0	0	0	0
56	NA	Plasma	EGFR WT	NA	8.8	-	-	0	0	0	0	0	0	0	0
57	NA	Plasma	EGFR WT	NA	2.8	-	-	0	0	0	0	0	0	0	0
58	NA	Plasma	EGFR WT	NA	8.7	-	-	0	0	0	0	0	0	0	0
59	NA	Plasma	EGFR WT	NA	9.2	-	-	0	0	0	0	0	0	0	0
60	NA	Plasma	EGFR WT	NA	46.4	-	-	0	0	0	0	0	0	0	0
61	0	Plasma	L858R	NA	41.3	-	-	0	1.2	0	0	0	2.0	0	0
	83	Plasma	L858R, T790M	NA	13.3	-	-	0	26.2	1.5	0	0	29.8	11.1	0
	461	Plasma	L858R, T790M	NA	28	-	-	0	40.1	29.9	0	0	65.4	53.6	0
62	0	Plasma	E19 delins, T790M	c.2235_2249del	30	-	-	40.1	0	9.9	0	48.5	0	17.6	0
	123	Plasma	EGFR WT	NA	105.3	-	-	0	0	0	0	0	0	0	0
	193	Plasma	EGFR WT	NA	50.7	0	0	0	0	0	0	0	0	0	0
	246	Plasma	E19 delins	c.2235_2249del	186	-	-	2.4	0	0	0	2.4	0	0	0
	560	Plasma	E19 delins, T790M, C797S	c.2235_2249del	46.7	-	-	34.0	0	6.8	6.9	33.3	0	5.8	2.7
	630	Plasma	E19 delins, T790M, C797S	c.2235_2249del	308	-	-	30.9	0	5.1	6.0	41.8	0	6	1.8
	663	Plasma	E19 delins, T790M, C797S	c.2235_2249del	226.7	-	-	18.4	0	2.8	3.2	24.5	0	4.7	1.2
63	0	Plasma	E19 delins	c.2240_2254del	29.3	-	-	13.2	0	0	0	13.7	0	0	0
	172	Plasma	E19 delins, T790M	c.2240_2254del	20	2.8	0.1	5.1	0	1.3	0	4.6	0	1.1	0
	205	Plasma	E19 delins, T790M	c.2240_2254del	62	-	-	5.5	0	1.1	0	7.6	0	1.0	0
	267	Plasma	EGFR WT	NA	46.7	-	-	0	0	0	0	0	0	0	0
	373	Plasma	E19 delins	c.2240_2254del	46.7	-	-	0.3	0	0	0	0.3	0	0	0
64	0	Plasma	E19 delins	c.2239_2251delinsC	9.3	-	-	18.6	0	0	0	11.4	0	0	0
	52	Plasma	E19 delins	c.2239_2251delinsC	12	-	-	32.3	0	0	0	28.6	0	0	0
	260	Plasma	E19 delins	c.2239_2251delinsC	37.3	3.9	0	5.2	0	0	0	5.2	0	0	0
	314	Plasma	E19 delins	c.2239_2251delinsC	13.3	-	-	21.2	0	0	0	21.5	0	0	0
	350	Plasma	E19 delins	c.2239_2251delinsC	32.6	-	-	15.9	0	0	0	14.4	0	0	0
	416	Plasma	E19 delins	c.2239_2251delinsC	84	-	-	3.5	0	0	0	3.7	0	0	0
	493	Plasma	EGFR WT	NA	93.3	-	-	0	0	0	0	0	0	0	0

NA: not applicable.

-: not available.

**Figure 2:**
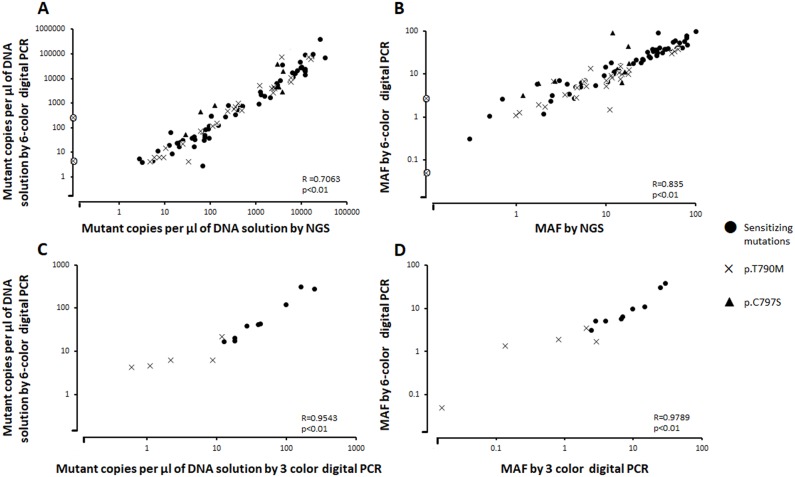
**(A)** Number of copies per μl of DNA solution and **(B)** Mutant allele fraction (MAF, %) of *EGFR* sensitizing and resistance mutations in tumor and plasma samples of NSCLC patients measured by 6 color digital PCR and by NGS. **(C)** Number of copies per μl of DNA solution and **(D)** MAF (%) of *EGFR* sensitizing and resistance mutations in 10 plasma samples measured by 3 color and 6 color digital PCR. Additional “o” mark in A) and B): *EGFR* T790M mutation detected by the 6-color digital PCR assay but not detected by NGS.

When considering all mutations, the six-color digital PCR assay revealed a mutant allele fraction (MAF) of 0.05% to 53.9% in plasma samples, representing a range of 61.3 to 10763 *EGFR* mutant copies per mL of plasma. Concentration of mutant copies per μl of DNA solution and MAF in tumor and plasma samples measured by 6-color digital PCR were compared to those measured by NGS and displayed a good correlation with a significant Pearson coefficient R of 0.7611 and 0.8434 respectively (P < 0.01) (Figure 2). The NGS analysis on patients indicates that the six-color digital PCR assay was able to detect 12 in-frame deletion-insertions in exon 19 of variable genomic sequences (Table 1).

Fifteen plasma DNA extracts were also tested in the same analytical conditions using 3-color digital PCR assays previously developed [21] and the results were compared to that of 6-color digital PCR and NGS. The 3-color digital PCR assay detected 9 activating mutations, also detected by 6-color digital PCR and NGS. The T790M resistance mutation was detected in 5 samples by both 3-color and 6-color digital PCR while this mutation was detected in 4 out of 5 samples using NGS. Comparison of concentration of mutant copies per μl of DNA solution and MAF in plasma samples measured by 6-color and 3-color digital PCR displayed a good correlation with a significant Pearson coefficient R of 0.9543 and 0.9789 respectively (Figure 2).

### Monitoring of *EGFR* mutations in longitudinal samples

**Figure 3:**
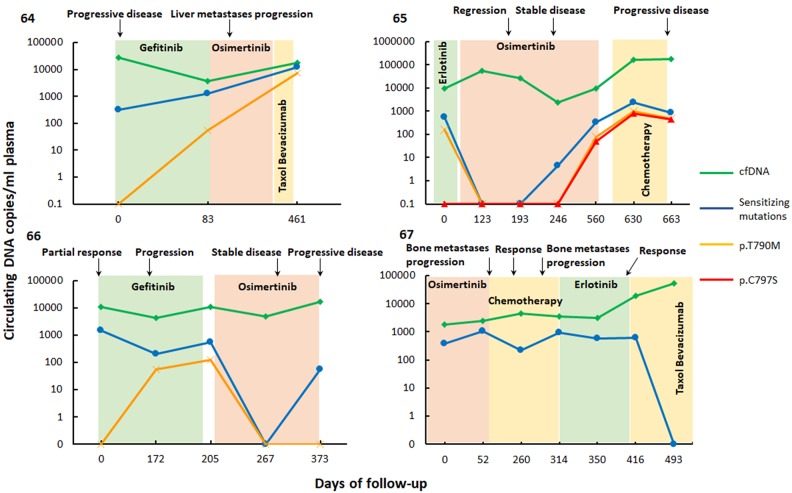
Quantification of the most prevalent sensitizing and resistance *EGFR* mutations and cell-free (cf) DNA levels in longitudinal plasma samples of metastatic NSCLC patients 61, 62, 63, 64 using six-color digital PCR Colored regions indicate periods of chemotherapy. Radiological assessments of patient response are indicated above the graphs.

To evaluate six-color digital PCR for the monitoring of *EGFR* mutations in NSCLC patients under treatment, the plasma samples of 4 patients, drawn at various timepoints, were analyzed (Figure 3). The concentration of total cell-free DNA, as well as the fraction of circulating tumor DNA, and lines of therapy and radiological assessments, were recorded. Overall, the level of circulating tumor DNA reflected well the course of the disease. Patient 61, treated successively by a first and a third-generation tyrosine kinase inhibitor, displayed a disease progression which correlated with an increase in levels of *EGFR* p.L858R (sensitizing) and *EGFR* p.T790M (resistance) mutations in cfDNA. Patient 62 was initially treated using first-generation TKI but was found to harbor *EGFR* p.T790M resistance mutation, and therapy was thus changed to third-generation *EGFR*-TKI osimertinib. Disease regression was consequently observed, supported by an initial decrease of both *EGFR* exon 19 deletion and p.T790M mutation plasma levels. However, further follow-up at later timepoints revealed increasing levels of both *EGFR* p.C797S and p.T790M resistance mutations, with disease progression later confirmed by radiological observations. Patient 63, initially treated with gefitinib, displayed T790M-mediated resistance to this first-generation TKI, and exhibited disease progression. Treatment with third-generation *EGFR*-TKI osimertinib was followed by a decrease of *EGFR* exon 19 deletion and p.T790M resistance mutation plasma levels. However, the last timepoint assayed revealed an increase of *EGFR* exon 19 deletion plasma levels, concomitant with disease progression, as observed by radiological assessments. Patient 64 was treated unsuccessfully with different therapeutic approaches, and plasma levels of activating mutations remained high, until the administration of Taxol, in association with Bevacizumab, which was followed by a clear decrease of *EGFR* exon 19 deletion plasma level to undetectable levels.

## DISCUSSION

The development of multiplexed assays is usually considered a cumbersome task in PCR. This is mostly due to the difficulty of matching PCR efficiencies for all primers/ probes systems involved as well as dealing with potential interactions/ competitions between detection systems. In a multiplexed digital PCR assay, PCR efficiencies may differ amongst the different detection systems, yet this will not necessarily affect quantification of targets. Since the reactions occur in separate partitions (here droplets), PCR efficiency can be monitored for each individual partition and any negative impact on PCR efficiency can be easily identified as partitions of lower fluorescence, which can still be accurately classified as either positive or negative. However, to date, multiplex digital PCR assays involving three or more targets remain seldomly reported due to the limited number of fluorescence channels available on existing platforms. To increase multiplexing power, several strategies have been be developed. One such strategy relies on separately identifying multiple targets in individual detection channels by means of intensity-multiplexing [19, 29]. Although this represents a very interesting strategy, which takes advantage of a unique feature of digital PCR, robust population quantification may prove difficult in presence of large quantities of DNA, or DNA of lesser quality. It is also possible to combine probes of identical sequences but labelled with different fluorophores to generate unique populations of double- (or multiple-) positive droplets, as was previously performed for a multiplex *KRAS* mutations detection assay in a two-color digital PCR system [29]. However, it is important to note that this last strategy relies on the presence of low quantities of targets, or alternatively a high number of partitions, to achieve low partition occupancy, as increasing DNA concentration will otherwise lead to an increase in random co-encapsulation of different DNA targets, which could yield significant noise in such assays.

In this study, we implemented a groundbreaking 6-color digital PCR system, which enabled monitoring of *EGFR* most prevalent mutations in NSCLC using a unique assay. By combining previously developed multiplex *EGFR* assays [22, 27], as well as adding other *EGFR* mutations relevant to NSCLC patient monitoring, we developed an assay capable of detecting 5 sensitizing mutations (p.L858R, p.L861Q, p.G719S, p.G719A, p.G719C), 12 different delins in exon 19, and 2 resistance mutations (p.T790M and p.C797S) in a single reaction. Mutations detected were not all individually identified in this assay, but instead functionally grouped in separate detection channels according to current knowledge regarding treatment efficacy prediction.

The sensitivity of detection of this assay was tested on serial dilutions of mutant DNAs, and could reach 0.0975% of mutant DNA sequence in a background of 10000 wild-type DNA sequences depending on the mutation targeted. Those values are comparable to that observed in other studies targeting *EGFR* mutations, with the assay exhibiting a sensitivity deemed clinically relevant for the detection of *EGFR* mutations in plasma samples [18, 19, 23, 24, 26].

Technology-specific limitations aside, sensitivity of detection is first and foremost conditioned by the total number of wild-type DNA fragments assayed. When characterizing the 6-color digital PCR assay on serial dilutions of DNA standards, we used 10,000 wild-type DNA copies per reaction as representative of DNA amounts available when assaying patient cfDNA samples. However, as discussed for patient 37 below, some samples may contain a much higher concentration of DNA, for example due to higher levels of cfDNA in plasma, or to DNA preparation protocols that include concentrating the extracted cfDNA. As LOB (and thus LOD) is not expected to correlate with wild-type DNA amounts [29], the measured mutant allelic fraction may be lower for such samples than evaluated with our experiment using serial dilution of DNA standards. In clinical settings, preanalytical and analytical parameters such as the volume of plasma processed, DNA extraction protocols and amount of DNA assayed must thus be carefully optimized and monitored to reach the sensitivity required for specific clinical applications.

This 6-color digital PCR assay was also successfully validated on DNA extracted from tumor and plasma samples of late stages NSCLC patients under treatment, and enabled cfDNA monitoring which revealed mutational loads consistent with disease evolution. However, when comparing results obtained with 6-color, 3-color dPCR and NGS on tumor and plasma samples, we found two samples for which dPCR identified the presence of p.T790M resistance mutations, which were not detected by NGS. For patient 37 plasma sample, the results discrepancy between technologies is easily explained by the high concentration of cfDNA in this sample, which enabled quantification of *EGFR* pT790M fractions of 0.02-0.05% with 3-color and 6-color digital PCR respectively, whereas the amount of DNA assayed by NGS could have only enabled at best the detection of a 0.1% MAF (10 ng assayed, equivalent to 3030 haploid genome copies). The lack of detection of the *EGFR* p.T790M mutation for patient 2 tumor sample by NGS is more puzzling however, since 6-color digital PCR quantified a 2.8% MAF, well within the detection range otherwise exhibited by NGS in this study.

As knowledge about NSCLC theragnostic and prognostic markers progresses, and new therapies emerge, clinicians’ needs for detecting specific subsets of genetic alterations may evolve. This 6-color digital PCR assay can be flexible and evolutive in terms of assay design. It could for example be envisioned to separately detect rare *EGFR* mutations such as p. G719A/C/S and p.L861Q (currently detected in the Cy-5 channel alongside p. L858R) and p.S768I, using one of the alternative multiplexing strategies described above. These mutations have been reported as less sensitive to first generation TKIs but displayed a favorable treatment response to second generation TKI afatinib [25], as such, their separate characterization could increase the patient's stratification value of this six-color digital PCR assay.

*EGFR* mutation testing has now been approved on plasma samples as a companion diagnostic for *EGFR*-TKIs by several governmental agencies worldwide. Crystal digital PCR exhibits advantages in term of sensitivity, cost and turnaround time which renders the 6-color multiplex *EGFR* assay characterized in the present study appropriate for such testing. This increase of the number of detection channels, combined with the exquisite sensitivity and precision of digital PCR, and a multiplexing strategy based on clinician diagnostic needs, yields promising avenues for tumor genotyping and monitoring in oncology. While this assay specifically addressed *EGFR* mutation status and monitoring in NSCLC patients, we believe it can also be used as a blueprint for other assay developments in oncology.

## MATERIALS AND METHODS

### Patients

Digital PCR and NGS analysis were conducted on blood or tissue samples from eligible patients with advanced stage IV NSCLC treated at the Institut Gustave Roussy cancer center (Villejuif, France) from January 2011 to July 2017. All patients provided written informed consent for biomedical research and the institutional ethics committee approved the protocols (NCT02105168; NCT02666612).

### Sample collection and processing

Blood samples (10 ml) were collected in EDTA-K2 tubes (BD Vacutainer ± Beckton, Dickinson and Company, Franklin Lakes, NJ) and centrifuged for 10 minutes at 1000 g within 4 hours after blood draw. Plasma was collected and further centrifuged at 14,000 g for 10 minutes at room temperature. The supernatant was stored at −80°C until analysis. Circulating cell-free DNA (cfDNA) was extracted from 500μL to 5 mL of plasma using the QIAamp circulating nucleic acid kit (Qiagen, Hilden, Germany) according to manufacturer's instructions, and resuspended in 40 μL of AVE buffer. A real-time quantitative PCR Taqman™ assay targeting GAPDH was used to measure circulating cell-free DNA concentration. Tumor DNA was extracted from frozen biopsy and FFPE samples using the AllPrep DNA/RNA Mini Kit (Qiagen) and the Maxwell^®^ RSC DNA FFPE Kit on the Maxwell^®^ RSC Instruments (Promega, Charbonnières-les-Bains, France) respectively, according to manufacturer's instructions and quantified with Qubit 2.0 (Thermo Fisher Scientific, Illkirch, France).

### *EGFR* mutations screening by NGS

Next generation sequencing analyses were conducted as previously described [21]. Targeted sequencing libraries were generated using The Ion AmpliSeq Library kit 2.0 and the Cancer Hotspot Panel v2 (CHP2) according to manufacturer's instructions (Thermo Fisher Scientific, Illkirch, France). Following purification and quantification, equal amounts of each library were pooled, emulsified and PCR amplified with the Ion OneTouch 2 system using the Ion PGM TM Template OT2 200 Kit (Thermo Fisher Scientific, Illkirch, France). The enrichment was then performed with the Ion One Touch ES (Enrichment System) and the enriched Ion Spheres were loaded into a 316v.2 Ion Sequencing Chip. Sequencing data was analyzed using the Torrent Suite Variant Caller 4.2 software and reported somatic variants were compared to the reference genome hg19. All the variants identified were visually controlled on BAM files using Alamut Visual v2.8.x software (Interactive Biosoftware, Rouen, France). All the germline variants found in 1000 Genomes Project or ESP (Exome Sequencing Project database) with frequency >0.1% were removed. All somatic mutations were annotated, sorted and interpreted by an expert molecular biologist according to available databases (COSMIC, TCGA).

### Design of the six-color digital PCR panel

**Figure 4:**
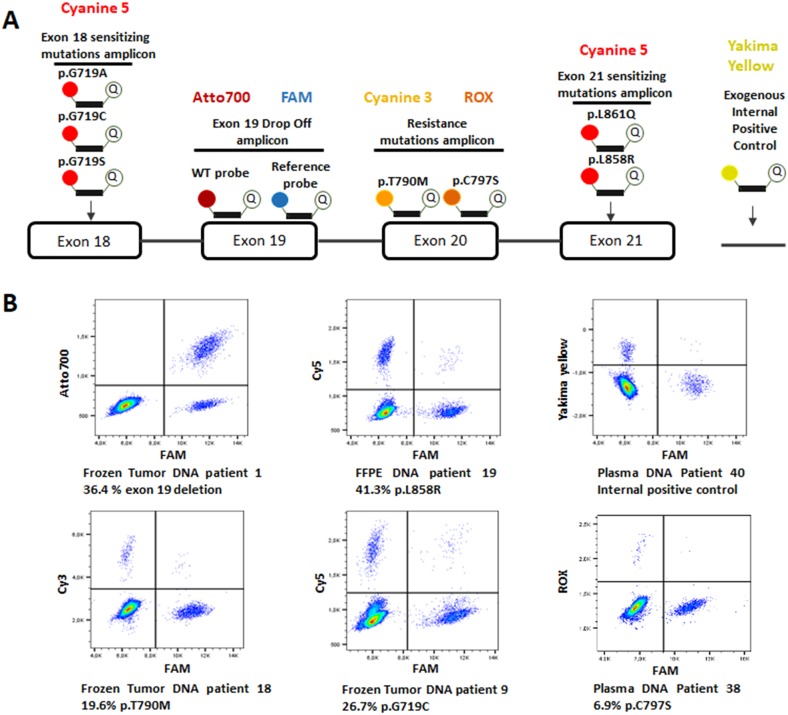
**(A)** Design of the six-color digital PCR assay. A total of 4 primer pairs were used to amplify 4 regions on *EGFR* exon 18, 19, 20 and 21. Six channels of fluorescence were selected to differentiate the targets of interest. Two probes respectively labelled with FAM and Atto700 were used to detect both the *EGFR* wild-type sequence and exon 19 delins in a drop-off assay. Cyanine 5 labelled probes were used to detect p.L858R/p.L861Q and p.G719A/C/S mutations. Cyanine 3 and ROX labelled probes detected the p.T790M and p.C797S resistance mutations respectively. Finally, a probe with a Yakima Yellow fluorophore was added to detect an exogenous DNA sequence which serves as an internal control of PCR amplification. **(B)** Six-color digital PCR results in 2D dot-plots from 6 tumor and plasma samples.

The six-color digital PCR panel for the detection of the most prevalent sensitizing and resistance *EGFR* mutations in NSCLC was designed by combining hydrolysis probes targeting specific *EGFR* mutations and a drop-off assay targeting the delins in *EGFR* exon 19 previously described elsewhere [25] (Figure 4A). This drop off assay uses one set of primers and two probes: a probe labelled with ATTO700, annealing to the region spanning the delins hotspot, termed the wild-type probe, and a reference probe, labelled with FAM, annealing upstream of the delins hotspot on the same amplicon, and termed the reference probe. If no deletion or insertion is present, both probes anneal to their targets on the amplicon, resulting in the partition fluorescing in both the ATTO700 and FAM channels. In contrast, in presence of delins in the targeted hotspot in exon 19, the wild-type drop-off probe cannot anneal, while the reference probe can still anneal to its target, resulting in partitions fluorescing in the FAM channel only (Figure 4B). Primers and Cyanine 5-labelled hydrolysis probes used for the detection of EGFR p.L858R (c.2573T>G) and p.L861Q (c.2582T>A) in exon 21 have been described previously [27]. The *EGFR* p.G719A (c.2156G>C), p.G719C (c.2155G>T) and p.G719S (c.2155G>A) in exon 18 and the *EGFR* p.T790M (c.2369C>T) p.C797S (c.2389 T>A) and C797S (c.2390 G>C) mutations in exon 20 were detected with primers and probes described in Table 2. All PCR assays generated amplicons shorter than 150 base pairs to be suitable for circulating cell-free DNA detection. Primers and probes were purchased from Eurogentec (Liege, Belgium). A universal exogenous qPCR positive control (Eurogentec, Liege, Belgium) using a Yakima Yellow-labelled hydrolysis probe was included in this six-color panel design. Sensitivity of detection of exon 19 delins between amino acids 746 and 750, p.G719A, p.G719C, p.G719S, p.L858R, p.L861Q, p.T790M and p.C797S mutations was tested in six-color experiments on serial dilutions of standard mutated DNA (Horizon Discovery, Cambridge, UK) and limit of blank (LOB) was measured on wild-type DNA (Bioline, London, UK).

**Table 2:** Primers and probes design for the detection of *EGFR* p.G719A, p.G719C, p.G719S, p.T790M and p.C797S

Name	Oligo type	5’fluorophore	Sequence	3’ modification
p.G719A Forward	Primer	NA	CCAACCAAGCTCTCTTGAGG	NA
p.G719A Reverse	Primer	NA	CCTTATACACCGTGCCGAAC	NA
p.G719A Probe	Hydrolysis probe	Cy5^®^	TGCTG+GCCTCCGGTG	BHQ-3
p.G719C Probe	Hydrolysis probe	Cy5^®^	TG+CTGTG+CTCCGGTG	BHQ-3
p.G719S Probe	Hydrolysis probe	Cy5^®^	TG+CTGAG+CTCCGGTG	BHQ-3
p.T790M p.C797S Forward	Primer	NA	GCAGGTACTGGGAGCCAAT	NA
p.T790M p.C797S Reverse	Primer	NA	GCATCTGCCTCACCTCCA	NA
p.T790M Probe	Hydrolysis probe	Cy3^®^	ATGAGCT+G+CA+T+GATGAG	BHQ-2
p.C797S c.2389 T>A Probe	Hydrolysis probe	ROX	CTTCGGCAGCCTCCTG	MGB eclipse^®^
p.C797S c.2390 G>C Probe	Hydrolysis probe	ROX	CTTCGGCTCCCTCCTG	MGB eclipse^®^

The + sign is a prefix designating a Locked Nucleic Acid (LNA) base.

NA: not applicable.

### Crystal™ Digital PCR detection of *EGFR* mutations from NSCLC patients

Six-color experiments were performed on a customized Naica Crystal Digital PCR system (Stilla Technologies, Villejuif, France). Plasma DNA and tumor DNA extracts were assayed using 2 and 1 replicates respectively. A replicate contained 3 μl of circulating cell-free DNA or 1 μl of diluted tumor DNA and was assembled in 25μl PCR mixtures using 1 X PerfeCTa Multiplex qPCR ToughMix (Quanta Biosciences, Gaithersburg, MD, USA), 250 nM Fluorescein (Sigma, Saint Louis, MO, USA), 1.25X qPCR Internal Positive Control (IPC) Yakima Yellow-BHQ-1 and 0.25 μl qPCR Internal Positive Control (IPC) DNA template (Eurogentec Liege, Belgium). Primer pairs final concentration for amplification of targets on exons 18 and 19 was 500 nM, and for exon 20 and 21, 750nM and 150 nM respectively. Hydrolysis probes were added at the following concentrations: 500nM for exon 19 reference, p.C797S (c.2389 T>A) and p.C797S (c.2390 G>C), 750nM for EGFR p.T790M (c.2369C>T), 75 nM for both p.L858R (c.2573T>G) and p.L861Q (c.2582T>A), 125 nM for p.G719A (c.2156G>C), p.G719C (c.2155G>T) and p.G719S (c.2155G>A) and 1 μM for exon 19 wild-type probe. A total of 0.25μl MunI restriction enzyme (ThermoFisher, Illkirch, France) was added to the PCR mixtures to fragment tumor DNA. Four PCR reactions were loaded per Stilla's Sapphire chip, compartmentalized in four chambers into 2D monolayers of droplet partitions and thermocycled using the Naica Geode instrument. Cycling conditions were 95°C for 10 minutes, followed by 50 cycles of 95°C for 30 seconds and 62°C for 30 seconds. Sapphire ships were imaged by fluorescence microscopy at 4X magnification using an inverted Nikon eclipse TI microscope (Nikon Instruments Europe, France) equipped with a motorized stage in the X, Y and Z axes, a Spectra X light engine (Lumencor, Beaverton, USA) and a DS-Qi2 camera (Nikon Instruments Europe, France). Filter sets (Optoprim, Paris, France) were selected for fluorescence readout in six distinct detection channels.

### Data analysis

A total of 6 × 9 images was acquired per chamber using the Nikon Eclipse-Ti microscope and assembled into large images using the NIS-Element software (Nikon Instruments Europe, France). Droplet identification and fluorescence measurements in each detection channel were performed using a modified version of Stilla's Crystal Miner software before being exported as CSV files. Spill-over compensation and gating of positive and negative droplet clusters were performed using FlowJo v10.0.8 (FlowJo LLC, Ashland, OR, USA). The number of copies of targets in each detection channel were derived from the fraction of positive partitions using Poisson statistics. The LOB was subtracted from the number of positive partitions measured for each patient sample tested to accurately calculate the concentration values. For the quantification of *EGFR* exon 19 delins, the calculation of the fraction of positive partitions was performed as follows: the number of FAM simple positive partitions was divided by the total number of FAM simple positives and FAM and Atto700 double negative population. The number of FAM and Atto700 double positive partitions containing mutant fragments co-encapsulated with wild-type fragments was not considered in the calculation as described previously [28].

## Abbreviations

EGFR: epidermal growth factor receptor
NSCLC: non-small cell lung cancer
TKI: tyrosine kinase inhibitor
FDA: Food and Drug Administration
cfDNA: cell-free DNA
LOB: limit of blank
MAF: mutant allele fraction
NGS: next generation sequencing.
