# Correction: *EGFR* C797S, *EGFR* T790M and *EGFR* sensitizing mutations in non-small cell lung cancer revealed by six-color crystal digital PCR

**DOI:** 10.18632/oncotarget.26686

**Published:** 2019-02-12

**Authors:** Jordan Madic, Cécile Jovelet, Julien Lopez, Barbara André, Jean Fatien, Isabelle Miran, Aurélie Honoré, Laura Mezquita, Benjamin Besse, Ludovic Lacroix, Magali Droniou

**Affiliations:** ^1^ Stilla Technologies, 1 Mail du Professeur Georges Mathé, Villejuif, France; ^2^ Plateforme de Génomique, Module de Biopathologie Moléculaire et Centre de Ressources Biologiques, AMMICa, INSERM US23/CNRS UMS3655, Gustave Roussy, Villejuif, France; ^3^ Ecole Polytechnique, Route de Saclay, Palaiseau, France; ^4^ Département de Biologie et Pathologie Médicales, Institut Gustave Roussy, Villejuif, France; ^5^ Département d’Oncologie Médicale, Gustave Roussy, Villejuif, France

**This article has been corrected:** The correct author name is given below:

**Laura Mezquita**

Original article: Oncotarget. 2018; 9:37393-37406. https://doi.org/10.18632/oncotarget.26446

